# *In vitro* retention of a new thermoplastic titratable mandibular advancement device

**DOI:** 10.12688/f1000research.6061.1

**Published:** 2015-02-26

**Authors:** Marc Braem

**Affiliations:** 1 Lab Dental Materials, University of Antwerp, Antwerp, 2610, Belgium; 2 Special Care Dentistry, University Hospital Antwerp, Edegem, 2650, Belgium; 3 Faculty of Medicine and Health Sciences, University of Antwerp, Antwerp, 2610, Belgium

**Keywords:** mandibular advancement device, obstructive sleep apnoea, oral appliance, retention force

## Abstract

Oral appliance (OA) therapy with a mandibular advancement device (OAm) is a non-invasive, alternative approach to maintaining upper airway patency. The main requirement for an OAm to be effective is the adequate retention on the teeth while the patient is asleep. We evaluated the retentive forces of a new low-cost, customizable, titratable, thermoplastic OAm (BluePro ^®^; BlueSom, France). Dental impressions and casts were made for one patient with complete upper and lower dental arches including the third molars and class II bite proportions. A setup based on Frasaco ANA-4 models was also used. Two protrusive positions of the mandible were investigated: 3 mm and 8 mm, representing respectively 25% and 65% of the maximal protrusion. The forces required to remove the BluePro ^® ^device from the carriers were recorded continuously over 730 cycles (=365 days, twice a day) to simulate 1 year of clinical use. At 8 mm protrusion the BluePro ^® ^device showed retentive forces of ~27N. There was a slight but non-significant decrease in retentive forces in the tests on the epoxified carriers which was not found on the ANA-4 carriers. There were no significant differences between the carriers as a function of protrusion. The BluePro ^®^ device tested in the present study possesses sufficient retention forces to resist initial jaw opening forces and full mouth opening forces estimated to be ~20N. It could therefore broaden the indications for use of thermoplastic OAms. It could provide a temporary OAm while a custom-made OAm is being manufactured or repaired. Patients could be provided with a low-cost try-out device capable of reliable titration, providing an indication of effectiveness and of patient acceptance of an OAm, although the effect of device shape and size on therapeutic outcome is not yet known. Finally it could provide an affordable OAm solution in resource-restricted healthcare settings.

## Introduction

Oral appliance (OA) therapy with mandibular advancement devices (OAm) is becoming the main non-invasive and alternative approach to continuous positive airway pressure. By advancing the mandible and tongue, upper airway patency is favoured during sleep thereby preventing upper airway collapse ^1^. Although many types of OAm are available ^2– 5^, each requires retention on the teeth to maintain protrusion of the mandible during sleep. Lack of retention causes loosening of the OAm and may result in reduced efficacy of treatment, patient complaints about poor fit and an increased risk of side effects ^4, 6, 7^.

The main disadvantages of custom-made OAm are not only the cost, but also, more importantly, the time required to manufacture the device, while the obstructive sleep apnoea (OSA)-patient is not being treated. A comparable situation arises when the OAm of a patient under treatment needs to be repaired in the dental laboratory. Furthermore, clinical practice demonstrates that patients are often not willing to leave the OAm for repair because they have become dependent on the device for a good night’s sleep.

The primary requirement for an OAm to be effective is the adequate retention on the teeth while the patient is asleep. Recently, *in vitro* testing of such retentive characteristics of different types of OAm, including thermoplastic ones, was reported in the literature ^8^, demonstrating that generalization of the retentive characteristics with respect to the design and type of an OAm is not pertinent.

The present report compares the retentive forces of a novel customizable titratable thermoplastic low-cost OAm to other devices reported previously in the literature.

## Materials and methods

### Supporting tooth arcs

**Figure 1.**
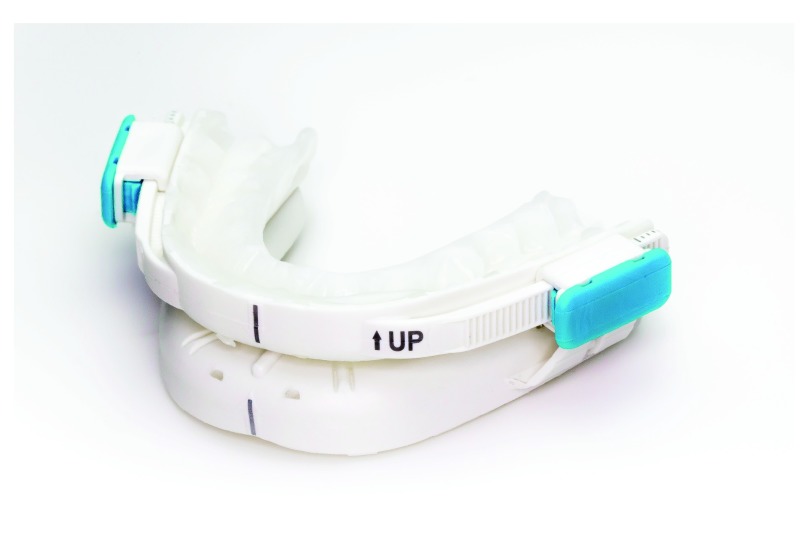
BluePro ^®^ device.

An experimental customizable titratable thermoplastic oral appliance commercialized for use by healthcare professionals under the name BluePro ^®^, was tested (BlueSom, France) ( Figure 1).

Dental impressions and casts were made (Impregum Penta Soft Medium, 3M ESPE AG, Germany) for one patient with complete upper and lower dental arches including the third molars and Class II bite proportions. A setup based on Frasaco ANA-4 models (Frasaco, Germany) was also made.

The protrusion to be incorporated into the BluePro ^®^ was measured from the vestibular rim of the incisive edge of the upper left central incisor, in a horizontal plane up to the lingual rim of the incisive edge of the lower left central incisor. These distances represent clinically relevant protrusive positions as noted during actual OA therapy in patients. Two protrusive positions were investigated in the present tests: 3 mm and 8 mm of protrusion, representing 25% and 65% of the maximal protrusion in the particular patient.

### Sample production

Samples of the BluePro ^®^ were fitted onto the epoxified casts (n=10 for each protrusion) as well as on the ANA-4 models (n=10 only for 8mm protrusion, see further) according to the manufacturer’s instructions (BluePro ^®^ user leaflet). The exact procedure was as follows:

Boiling water was poured into a flat-bottomed polypropylene box. The upper and lower parts of the BluePro ^®^ were then placed into the hot water until the inner material in both parts became fully transparent. A timer was started and the upper part was taken out of the water and put to one side to cool down for 30s. The same procedure was then repeated for the lower part and the upper part was immediately shaped onto the upper carrier, either the epoxified cast or ANA-4 carrier, depending on the test required.

The carrier was pressed firmly into the upper part of the BluePro ^®^, sliding it a bit forward to create an optimal fit into the tray of the BluePro ^®^. Finger pressure was then applied and the softened material on the inside of the tray was immediately flattened. The softened material was also pressed into shape at the outside of the tray to fit it tightly around the carrier. Both the carrier and the BluePro ^®^ were put on a flat table, the BluePro ^®^ facing down, and a dead weight was placed on top to simulate gentle biting and to immobilize the device in the required position, without popping or creeping out. This procedure was completed within 30s, then was repeated for the lower jaw.

Both parts were allowed to cool down for 5 min. The thermoplastic material inside the tray was then checked to see if it had become cold and opaque again and the BluePro ^®^ was removed from the carrier and rinsed in cold water. If necessary, sharp edges of superfluous softened material were removed, as well as any material that had crept into the rails or locks. The device was then dried and stored in the original packing at room temperature prior to testing.

### Sample mounting

The casts and ANA-4 models were mounted in average value inclination of the occlusal plane and centred in a hydraulic cyclic test machine (Dartec HC10, Testbench Dartec 9600 Controller; Dartec, UK) as described previously ^8^.

When mounting the casts, stubs were added to these to facilitate removal and reinsertion. Both upper and lower casts were mounted at a fixed position, giving a protrusion of 3mm or 8mm, depending on the test.

When mounting the ANA-4 carriers into the fatigue machine, first a bite-registration was made with silicone on a plastic spoon with a protrusion of 8mm compared to the habitual occlusion. Using this bite registration as a reference, the ANA-4 carriers were fixed in a reproducible way so that all BluePro ^®^ samples were tested in an identical protrusion. Due to technical limitations of this version regarding the length of the rim for the clasps, it turned out that the 3mm protrusion could not be tested. Next, for the fitting of the OAm-TP on the ANA-4 carriers a specific procedure was followed.

**Figure 2.**
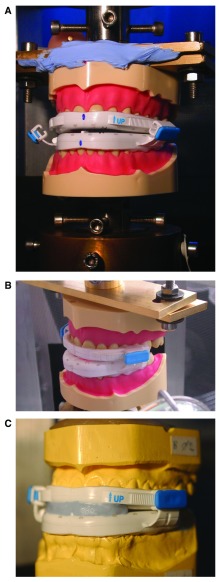
Casts and ANA-4 models used to test the retentive characteristics of the BluePro ^®^ device. ( **A**) positioning of the BluePro ^®^ device on the ANA-4 carriers that are connected to the pistons of the servo-hydraulic fatigue machine, prior to tightening of the titration clips; ( **B**) situation after tightening of the titration clips, joining the upper and lower part of the BluePro ^®^ device; ( **C**) identical situation as in ‘ **B**’ but on the epoxified gypsum carriers.

First the upper and lower parts of the BluePro ^®^ were inserted onto the carriers but without interconnecting ( Figure 2A). The upper carrier together with the OAm could now move up and down freely as the piston moved. The lower model stayed fixed to the bottom of the machine. The upper carrier with the BluePro ^®^ was then lowered until it touched the lower carrier establishing the zero-position calibration for the stroke. Next, both the upper and lower parts of the OAm were connected by sliding the locks onto the rails, but without locking and firmly seated thereafter by finger pressure. The locks were then closed and the BluePro ^®^ was positioned correctly in the machine for the given protrusion ( Figure 2B). In this position, there appeared to be a gap in the frontal region preventing the correct repositioning at reinsertion during the fatigue test. To avoid this phenomenon, a passive splint in silicone putty was inserted ( Figure 2C). The upper piston was moved upwards until the BluePro ^®^ came loose from either of the ANA-4 carriers and with the sample now hanging free and unloaded, the load (force) was set to zero so that both the stroke (displacement) and load (force) were calibrated correctly. The fatigue test was then started.

### Sample measurements

The forces required to remove the BluePro ^®^ from the carriers were continuously recorded during 730 cycles (=365 days, twice a day) at 35°C in a dry environment to simulate 1 year of clinical use. Data were stored in CSV format for export to Excel (Microsoft, USA, see Dataset 1 and Figshare Dataset).

The test was setup as a stroke-controlled test with a triangular waveform, thus the piston repeatedly moved up and down with a given stroke and frequency. The piston speed was 7.5mm/s and was kept constant during all tests. The stroke length was set to 7.5mm, back and forth from 0.0mm to 7.5mm. This stroke was chosen carefully so that the BluePro ^®^ came loose from the carrier, although not completely so that re-entrance could be carried out without misalignment at reinsertion during the next loading cycle. The test frequency was 7.5mm/s at the optimal stroke, resulting in a test frequency of 0.5Hz.

Graphs of the waveforms were captured every 36 cycles, starting at cycle 10 and ending at cycle 694. On the load graphs, the point at which the BluePro ^®^ came loose from the carrier was detected since the load dropped to zero and stayed at zero for the rest of the upward trajectory. At this point the load needed to give a flat line at zero, also indicating that the load-channel had been calibrated to zero correctly.

 Measurements were compared using a paired Student’s t-Test (Statistica Release 6, StatSoft Inc. Tulsa, USA) at a significance level of 0.05.

## Results

The removal forces (N) expressed as a mean (± SD) for both protrusion positions (3 mm and 8 mm) at the beginning and end of the tests revealing the effect of test duration are shown in Table 1: there was a slight but not significant decrease in retentive forces for the tests on the epoxified carriers, not to be found for the ANA-4 carriers. There were no statistical differences between the different test conditions and carriers as a function of the protrusion for the BluePro ^®^.

**Table 1.** Retentive forces (N) with standard deviation (SD) at the start and end of the test cycles for both the epoxified casts and ANA-4 model supports.

Protrusion		Cast		ANA-4	
		**Cycle 10**	**Cycle** **694**	**Cycle 10**	**Cycle** **694**
3 mm	Mean (N)	26.6	24.2	n/a	n/a
	SD (N)	4.4	3.2	n/a	n/a
	n samples	10	10	n/a	n/a
8 mm	Mean (N)	25.7	24.3	27.4	27.4
	SD (N)	3.4	2.9	5.9	6.6
	n samples	10	10	10	10

**Figure 3.**
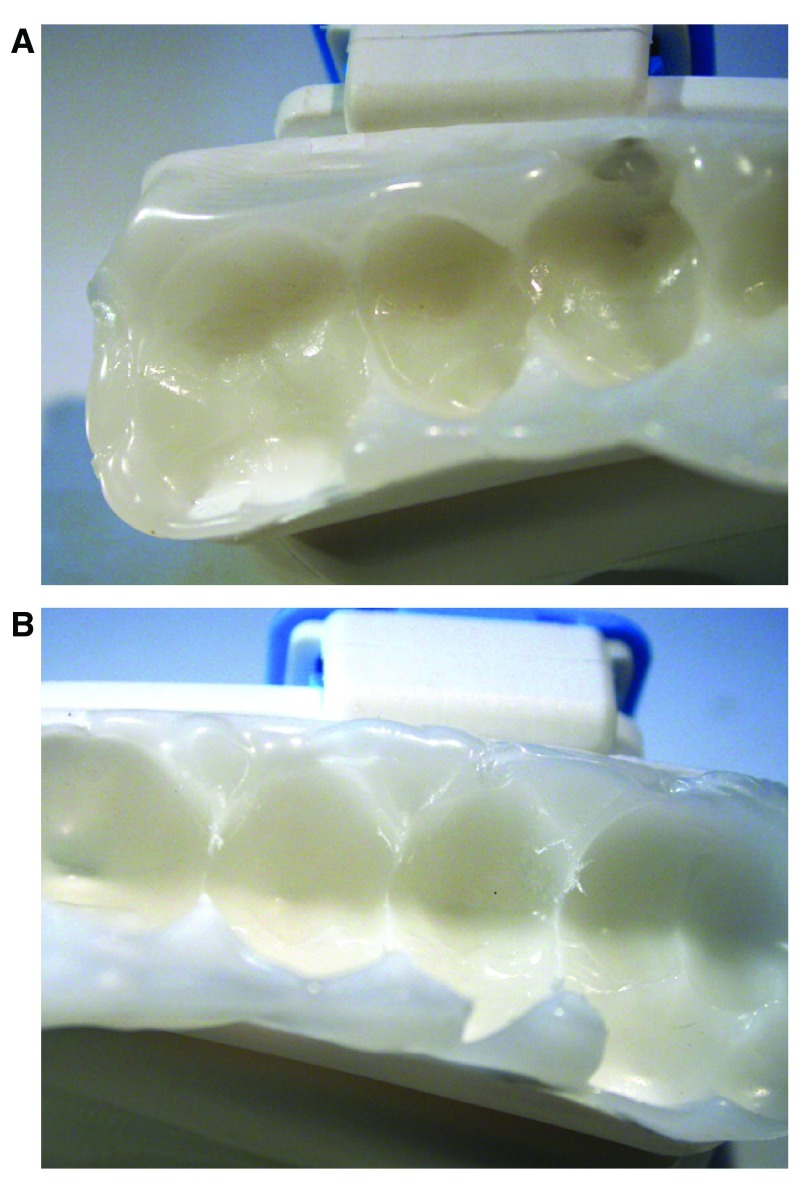
Wear particles and tear characteristics on the BluePro ^®^ device. ( **A**) Almost none to ( **B**) moderate signs of wear due to the repetitive loading, presenting itself under the form of small wear particles that are torn off the thermoplastic material as a consequence of the cyclic loading.

Inspection of the inner surfaces of the test samples revealed the presence of some wear particles and tear characteristics as shown in Figures 3A and 3B.

Raw data of MRA-fatigue tests Detailed information on each data file can be found in the txt file provided

Data of retentive forces of mandibular advancement device BluePro^®^ Sample parameters used for the different tests.

## Discussion

The setup used in this study extends the experimental setup used previously ^8^ by introducing a more standardized set of carriers with respect to tooth anatomy and shape of the tooth arcs. The results indicate that the measurements did not differ significantly between the two types of carriers, namely epoxified casts versus ANA-4 Frasaco models.

In OAm therapy for OSA it has not yet been defined to what extent variations in OAm design may affect clinical efficacy, side effects and patient compliance ^9^. Design features may also dictate retention of the OAm during sleep. Knowledge of such retentive characteristics is therefore essential for selection of an effective OAm for clinical use. It has previously been shown that it is feasible to evaluate *in vitro* retentive characteristics of monobloc types of OAm ^8^ and the results of the present study indicate that a duo-bloc OAm can also be tested in a fixed protrusive position, thereby resembling the monobloc setup. The present findings also indicate that, compared to the previously published results, the experimental OAm currently tested at 8mm protrusion showed retentive forces of ~27N as shown in Table 1. These findings further support the hypothesis ^8^ that introducing a soft thermoplastic body inside a more rigid tray improves retention of a thermoplastic type of OAm by improving rigidity and fit of the OAm. The present conclusion also supports recent findings with another prefabricated thermoplastic OAm that is also contained in a rigid shell ^7^.

In the present study, the retention forces measured for the thermoplastic OAm did not change significantly over time ( Table 1) and running-in wear and wear due to repetitive insertion and removal did not significantly influence the results. An increased amount of protrusion also did not cause higher retention forces ( Table 1), indicating that its retentive capacities are independent of mandibular protrusion for the protrusive range studied. Additional research and clinical reports are needed to further study these effects.

Jaw opening forces ^10^ at an interincisal distance of about 10 mm range from 2N to 9N and increase thereafter to an average of 19.9 ± 4.5N at an interincisal distance of about 49 mm. Looking at these values from the literature, it becomes clear that the thermoplastic OAm tested in the present *in vitro* study possesses sufficient retention forces to resist initial jaw opening as well as full mouth opening forces.

Often customizable thermoplastic OAms are less expensive than custom-made appliances in the dental technical lab. This additional element could broaden the indications for use of such devices. Since titration is now considered an inevitable prerequisite for obtaining success in OA treatment, better selection of appropriate patients for OAm therapy is essential. One approach to the selection of patients could be to provide the patient with a low-cost try-out device that is capable of reliable titration. Such a diagnostic tool would not only provide an evaluation of the effectiveness of an OAm in an at-home situation, but would also help check whether or not the patient is willing to use an OAm. It could also provide a temporary OAm while a custom-made OAm is being manufactured or repaired. Finally it could bring an OAm solution to certain resource-restricted healthcare settings in which custom-made appliances are unaffordable. Although these indications seem promising, it should be noted that some elements regarding the design of an OAm and its effects are not yet known. This includes the bulkiness of the OAm and its effect on the position of the tongue for example.

## Data availability

F1000Research: Dataset 1. Raw data of MRA-fatigue tests, 10.5256/f1000research.6061.d43120 ^11^

*Figshare*: Data of retentive forces of mandibular advancement device BluePro ^®^. 10.6084/m9.figshare.1306562 ^12^
