# Supplementary material for: In vitro retention of a new thermoplastic titratable mandibular advancement device
Source: F1000Res. 2015 Feb 26;4:56. [Version 1] doi: 10.12688/f1000research.6061.1 (PMC4392831; doi:10.12688/f1000research.6061.1)
Supplement: Peer review history

# Peer review: Referee response for version 1

**DOI:** 10.5256/f1000research.6489.r7832

**Published:** 2015-03-30

**Authors:** Julia Cohen-Levy

**Affiliations:** 1 Private Practice, Paris, France

This is a well designed *in-vitro *study, supported with adequate experimental data and statistical analysis. Results are accurately presented and discussed, in a clear and concise manner. The conclusions are justified on the basis of the presented results.

Some minor revisions could be positively added to the manuscript, in the discussion part. The flaws of the ' *in-vitro*' study of the retention of a thermoplastic oral device should, in my opinion, be also discussed as the human mouth motion has very little to do with the testing machine, in relation to lateral movements of the mandible. The influence of saliva, and maybe of mouth temperature might have an influence on the material properties with time.

I have read this submission. I believe that I have an appropriate level of expertise to confirm that it is of an acceptable scientific standard.

# Peer review: Referee response for version 1

**DOI:** 10.5256/f1000research.6489.r7860

**Published:** 2015-03-05

**Authors:** Timothy Quinnell

**Affiliations:** 1 Respiratory Support and Sleep Centre, Papworth Hospital NHS Foundation Trust, Papworth Everard, UK

The title accurately conveys the article’s content.  The abstract is a good summary of  the work being reported.  The author clearly describes the application of an *in vitro* method for measuring the retentive forces of mandibular devices, to a new titratable thermoplastic device.  This novel method was recently reported by the author and colleagues (citation 8).  It seems to be a useful *in vitro *tool for assessing the ability of mandibular devices to resist displacement by jaw opening – an important failing of many thermoplastic devices is poor oral retention.  The technique naturally has its limitations, which have been discussed in detail in citation 8.  It would be worth the author briefly referring to them in this article including: lack of lubrication; linear carrier movement  vs. non-linear, ‘least resistance’ pathway of jaw opening *in vivo*; the use of carriers from only 1 patient.  It is not clear how 730 cycles represents a year’s use.  If it refers to jaw opening at the end of the night to remove the device then should this not be 365?  Nonetheless the data are credible and provide useful insight (alongside citation 8) into the varying in vivo retentive properties of different devices; and device durability; and further demonstrate a potentially straightforward means of *in vitro* OA testing. The protrusions used were clinically relevant.  I agree with the author’s conclusions, that the results suggest thermoplastic OAms (with adequate retentive properties) could have a role in: titration; temporary custom made device substitution; and longer term treatment.  However I disagree that titration is now a prerequisite for successful OA treatment, because there is strong evidence that non-titratable devices do work ( Thorax 2014;69:10 938-945); and the superiority of titration has not been proven by rigorous head-to-head trials.  Finally, device effectiveness depends as much on comfort and tolerability (influencing adherence) as on oral retention.

I have read this submission. I believe that I have an appropriate level of expertise to confirm that it is of an acceptable scientific standard.
